# Effects of two non-drug interventions on pain and anxiety in the nursing process of burn patients: a literature review with meta-analysis

**DOI:** 10.3389/fresc.2024.1479833

**Published:** 2024-10-29

**Authors:** Wei Zhang, Xiaona Sui, Lingling Zhang, Liping Zhang, Huilan Yan, Shuangshuang Song

**Affiliations:** ^1^ Department of Burn and Plastic Surgery, Shandong Provincial Hospital Affiliated to Shandong First Medical University, Jinan, China; ^2^ Department of Stomatology, the 960th Hospital of People’s Liberation Army of China (PLA), Jinan, China

**Keywords:** burn, VR, music therapy, pain, anxiety

## Abstract

**Background:**

Burns are a global health issue causing significant mortality and high medical costs. Non-pharmacological interventions such as music therapy and virtual reality (VR) therapy have shown potential in alleviating pain and anxiety in burn patients. This study systematically evaluates the impact of these interventions using a network meta-analysis.

**Methods:**

A systematic review and network meta-analysis were conducted according to PRISMA 2020 guidelines and registered in PROSPERO (CRD42024566536). Searches in PubMed, Cochrane Library, Web of Science, and Embase up to November 22, 2023, identified randomized controlled trials (RCTs) involving music therapy or VR therapy in burn patients. The Cochrane Risk of Bias Tool (2.0) assessed study quality. Data were analyzed using StataMP-64 software.

**Results:**

Seventeen RCTs with 1,119 burn patients were included. Both music therapy and VR therapy significantly reduced pain and anxiety compared to control groups. Music therapy was more effective for pain reduction (SUCRA: 85.4%), while VR therapy was superior for anxiety relief (SUCRA: 79.5%).

**Conclusion:**

Music therapy and VR therapy effectively reduce pain and anxiety in burn patients. Integrating these interventions into burn care can enhance patient outcomes. Further research is needed to confirm these findings and optimize individualized treatment plans.

**Systematic Review Registration:**

https://www.crd.york.ac.uk/prospero/, PROSPERO (CRD42024566536).

## 1 Introduction

Burns are injuries to body tissue caused by exposure to various sources of heat, electricity, chemicals or radiation. These sources include hot liquids such as water, oils and soups, as well as steam, hot gases, flames, molten metals such as iron, aluminium and copper, and their liquid slags (1). Burns are a global public health problem, accounting for approximately 180,000 deaths each year (2, 3). Direct medical costs of burns can vary widely, but are typically high. In addition, indirect costs such as lost wages, long-term care for disability and psychological trauma, and invested family resources can further exacerbate the social and economic impact of burn injuries (2). Technological advances in burn injury treatment have led to declining mortality rates and extended life expectancy. However, previous studies have shown that burns can result in sequelae that reduce life satisfaction for the affected individual (4–7).

Burn patients frequently experience intense pain during treatments such as debridement and dressing changes, as well as during rehabilitation exercises (8). Patients often face emotional challenges, including anxiety and fear. Hence, it's essential that we bolster nursing interventions to alleviate patients’ suffering and anxiety, thereby enhancing therapeutic outcomes. These interventions are therefore very important in burn care and have been associated with improved survival rates in burn patients, a testament to advances in burn surgery (9, 10). Clinically, we usually divide interventions into drug interventions and non-drug interventions, and two common methods of the latter are music therapy and virtual reality (VR).While physicians are primarily responsible for administering pharmacological interventions, some non-pharmacological interventions can only be performed by nursing staff (11). Non-pharmacological interventions are important to the nursing process. A burn care guideline mentions non-pharmacological treatments, such as distraction, multimodal distraction (MMD), computer-based distraction, music-based distraction, virtual reality, hypnosis, and others (12).

The advantages of music therapy in the rehabilitation process of burn patients are mainly reflected in emotional support and psychological relaxation (13). It helps patients relieve anxiety and depression by providing positive emotional experiences and guided relaxation, while distracting attention from pain and reducing pain (14). Music therapy is personalized, that is, it can be customized according to the patient's preferences, is low-cost and easy to operate (15, 16).

Virtual reality (VR) therapy uses immersive technology and multi-sensory stimulation to provide a novel rehabilitation aid for burn patients (17). It distracts patients from pain and enhances a sense of control by creating an attractive virtual environment, and may be used to simulate real-life scenarios to help patients gradually adapt and overcome fear (18). VR therapy can also provide situational education and training, while allowing the medical team to evaluate the treatment effect through system-recorded data, bringing innovative treatment options to the field of burn rehabilitation (19). The advantages of virtual reality therapy in the rehabilitation process of burn patients are personalized customization, immersive experience, safe control and pleasure, which help improve patients’ rehabilitation effects and quality of life (20, 21).

Specifically, VR can cause interference not only visually but also auditorily, while music therapy mainly causes interference in the auditory sense (22). In addition to the different ways of inducing interference during the intervention process, there are also some differences in the implementation of music therapy and VR. On the one hand, music therapy is relatively cheaper and more practical, while VR requires expensive equipment and additional operating expenses (23). This makes music therapy more popular in many cases, especially for institutions and individuals who want to intervene on a limited budget (20). However, virtual reality technology can also provide burn patients with a pleasant and interesting rehabilitation experience, increase the fun and motivation of the rehabilitation process, and thus promote the patient's rehabilitation progress (24).

As two non-drug treatment methods, music therapy and virtual reality (VR) therapy provide different sensory stimulation and psychological intervention in the rehabilitation process of burn patients (13). Music therapy affects emotions and psychological states through auditory pathways, focusing on emotional expression and emotion regulation, and has the characteristics of low cost and easy popularization (25). It combines humanistic care and art therapy to provide emotional comfort and psychological relaxation for patients. VR therapy uses multiple pathways to work, including vision, hearing, etc. Through intuitive perception, control and exploration of the virtual environment, patients can participate more actively, improve their initiative, and help strengthen doctor-patient cooperation (24). Incorporating cutting-edge technology into healthcare, virtual reality (VR) treatment methods exemplify the advancement in scientific and technological fields. Moreover, by integrating with medical practices, VR therapy enhances patient outcomes and delivers additional advantages. Although the cost is relatively high, it shows great potential in personalized treatment (22).

These two methods were selected for comparison because they each represent different treatment concepts and technical applications, and can provide a comprehensive evaluation of treatment effects (26). The popularity and acceptability of music therapy combined with the innovation and personalization of VR therapy can provide a more comprehensive understanding of their mechanisms of action, advantages and limitations in burn rehabilitation (21, 27, 28). Through comparison, medical professionals can provide patients with more personalized and effective treatment plans, while exploring the possible complementarity and synergy of the two treatment methods to improve rehabilitation effects (25).

Although selected controlled clinical trials have shown that these two interventions provide some benefit, the results are not uniformly clear. Therefore, further demonstration is required to establish the role and value of these two interventions in the burn care process. The purpose of this paper is to use the method of reticulated Meta-analysis to collect relevant studies involving music therapy and VR interventions in the process of burn care. The aim is to systematically evaluate the impact of the two interventions on relieving pain, anxiety and other factors arising during the treatment process of burn patients. This will provide valuable decision-making basis for providing patients with comfortable treatment and improving their prognosis.

## 2 Methods

The study design was a systematic review that included a meta-analysis and was developed on the basis of the Preferred Reporting Items for Systematic Reviews and Meta-Analyses (PRISMA) 2020 guidelines (29). The study protocol was registered in the PROSPERO database (Registration number: CRD42024566536).

### 2.1 Search strategy

The search strategy was designed by two independent researchers, with a third researcher making decisions in cases of disagreement. Relevant articles were searched for supplements using computer searches of PubMed, Cochrane Library, Web of Science, and Embase databases from the date of construction to 22 November 2023, as appropriate. The search strategy was based on the following medical subject headings: “Burns”, “Music therapy”, and “Virtual Reality”. (Supplementary Table S1)

### 2.2 PICOS and inclusion/exclusion criteria for the study

Patients (P): Burn patients, regardless of burn area, age, race, gender, underlying disease, etc., regardless of treatment stage.

Intervention (I): “Music therapy” or “VR treatment”.

Comparison (C): Standard care without intervention.

Outcomes (O): Our primary outcomes of interest are changes in pain levels and symptoms of psychological conditions such as anxiety and relaxation. The secondary outcome is basic vital signs (including heart rate, respiratory rate and blood pressure).

Study Design (S): Only randomized controlled trials (RCTs) were included in this review.

Inclusion Criteria:(1) randomized controlled trial (RCT); (2) patients of any age, gender or ethnicity who had suffered burns, regardless of their severity, were included in the review;(3) with applied “Music therapy” or “VR treatment”; (4) with measured pain or anxiety outcomes.

Exclusion Criteria:(1) study types other than RCTs (quasi-randomized trials, non-randomized trials, observational studies, case reports, abstracts, or letters from the review.); (2) burn patients with hearing impairment, cognition impairment or communication disorder.

### 2.3 Outcomes

#### 2.3.1 Primary outcomes

• Pain, which was measured with visual analogue scales, numerical rating scales or other validated instruments.
• Symptoms of psychological, including anxiety and relaxation.

#### 2.3.2 Secondary outcome

• Basic vital signs (including heart rate, respiratory rate and blood pressure).

### 2.4 Literature screening and data extraction

Two researchers conducted an independent literature screening. They imported all retrieved literature into EndNote X9 software deleted duplicates, and initially screened literature by reading titles and abstracts. Finally, they screened literature that met the criteria by reading the full text based on the inclusion and exclusion criteria that had been previously developed. Relevant data such as author, publication year, participant characteristics, type of intervention, sample size, detailed information about intervention (like procedure, duration, frequency), type of scale were loaded onto a data extraction form. Decisions were made through discussion or with the involvement of a third researcher in cases of disagreement during the screening and data counting process.

### 2.5 Risk of bias assessment

The Cochrane Risk of Bias Tool (2.0) for RCTs was used to assess the quality of the included studies. The tool is based on five domains: bias in the randomization process, bias away from the intended intervention, bias in missing outcome data, bias in outcome measurement, and bias in selective reporting of results (30). The studies were categorised as “low-risk”, “high-risk” and “some concern”. Decisions were made through discussion or with the involvement of a third researcher in cases of disagreement during the literature quality assessment process.

### 2.6 Data synthesis and statistical analysis

We used StataMP-64 software to perform the Network Meta-Analysis. The data collected were summarised on the basis of continuous and dichotomous variables, and effect sizes were calculated using the mean difference (MD) for continuous variables and the odds ratio (OR) for dichotomous variables, both with 95% confidence intervals. The network evidence plot was created using the “Network map” command, which visualises the network relationships between different interventions. In addition, the area under the cumulative ranking curve (SUCRA) was used to rank and predict the strengths and weaknesses of each intervention, with the intervention with the greater area under the curve considered superior. Finally, to assess the presence of small sample effects and publication bias, we drew a funnel plot using the “netfunnel” command.

### 2.7 Ethical approval

As our research aggregates data from already published studies, we did not need to seek ethical approval for this meta-analysis. Each of the underlying studies adhered to the appropriate ethical standards and had received the required clearances from the responsible parties.

## 3 Results

### 3.1 Study and identification and selection

**Figure 1:**
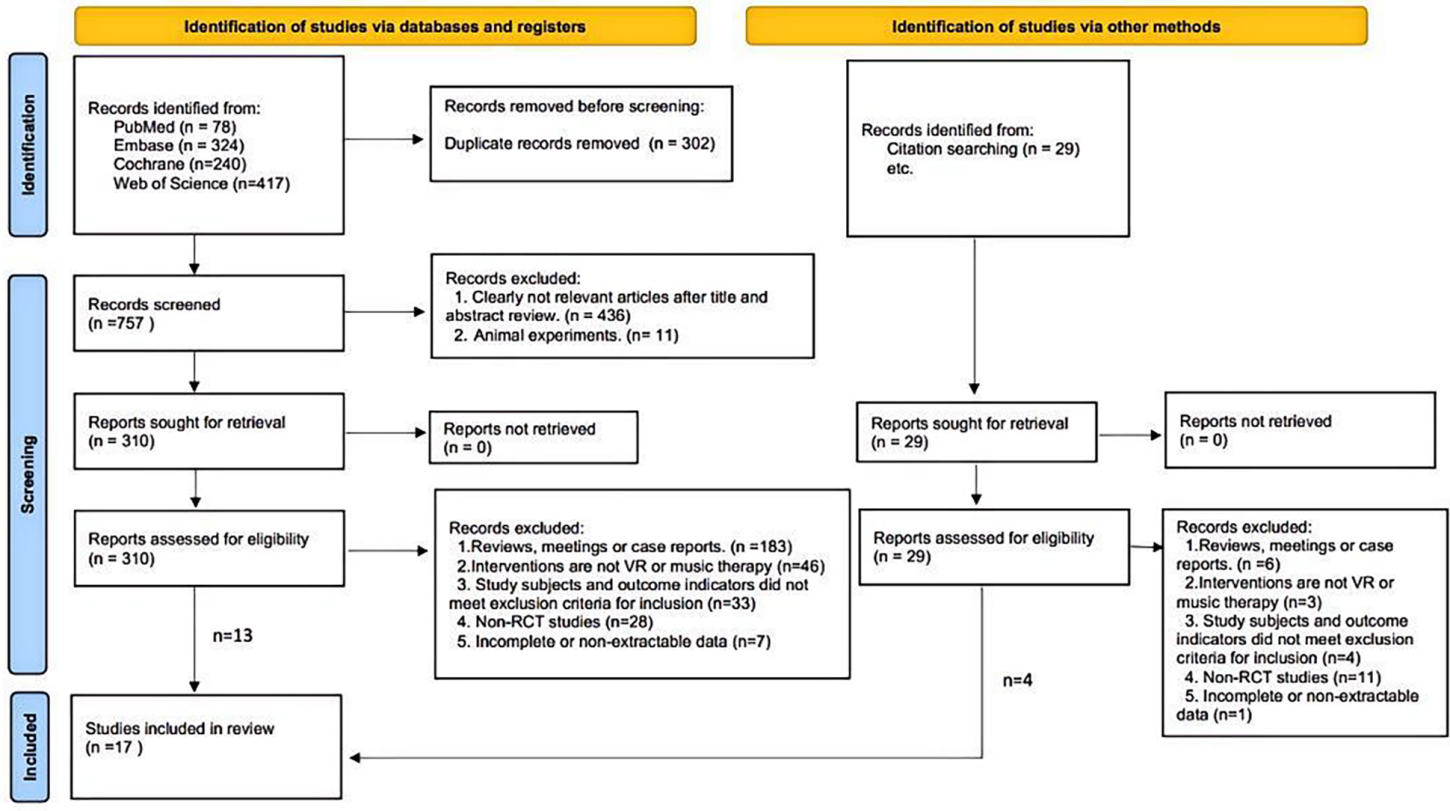
Flow diagram of literature selection.

A total of 1059 documents were retrieved from the electronic database, and an additional 29 documents were manually searched. After eliminating duplicates, the remaining 786 documents were screened for titles and abstracts, and 447 documents were again excluded. The remaining 339 documents were read in full and 322 documents were again excluded (for reasons including non-randomized trials, meetings, case reports, incomplete data, and failure to meet the interventions or outcomes included in this review), leaving a final remaining 17 documents to be included in this study (Figure 1).

### 3.2 Quality assessment of the included studies

**Figure 2:**
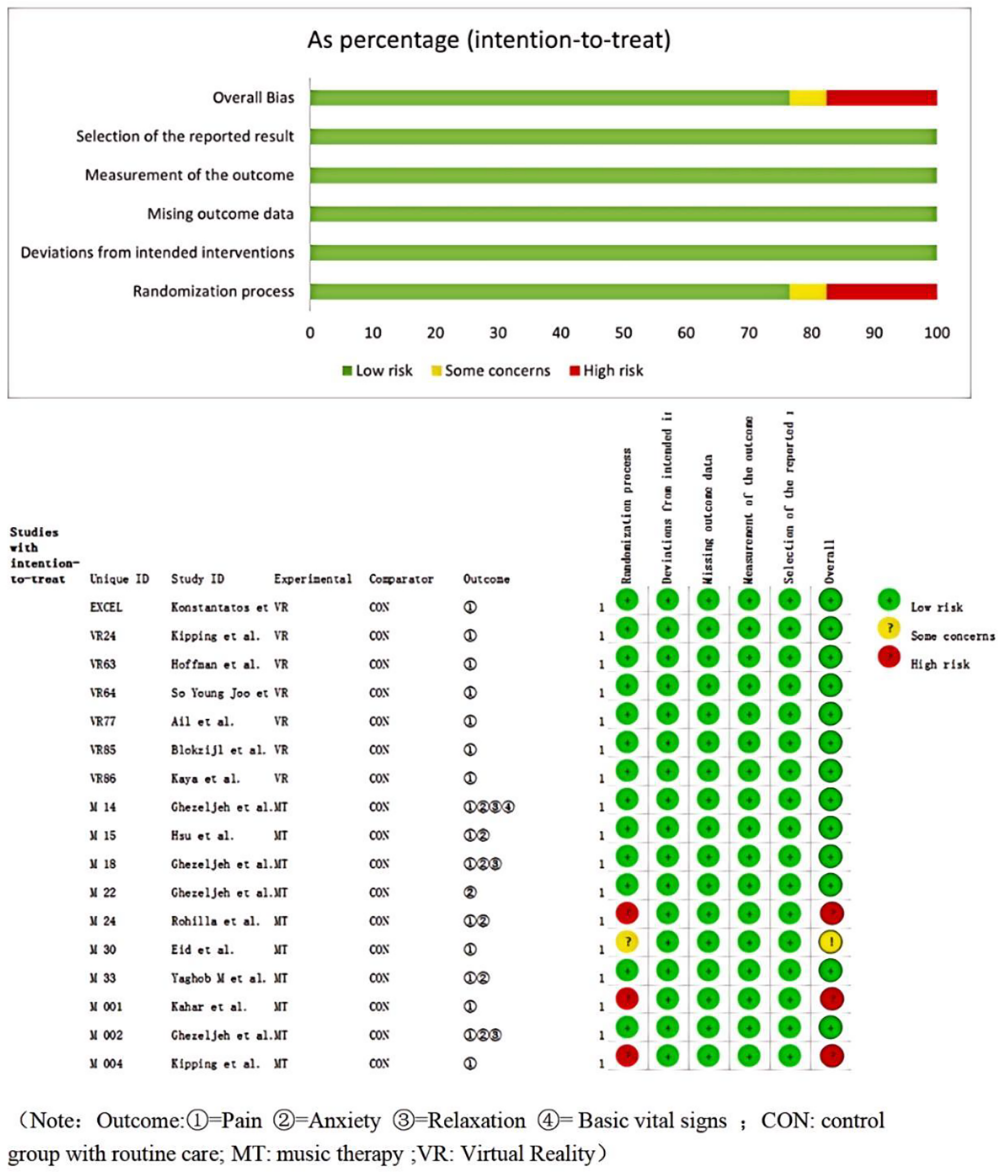
Cochrane risk of bias assessment results of the included studies.

The assessment of the quality of the literature indicates a low risk of bias for both individual papers and the literature as a whole. The 17 relevant papers included in the study were of good quality and were considered acceptable for inclusion (Figure 2).

### 3.3 Characteristics of the included studies

In total, we included studies from 17 randomised controlled trials, which included 1119 burn patients (including inpatients and outpatients). Interventions in the control group included music therapy (ten studies) (31–40), and virtual reality therapy (seven studies) (41–47). Sixteen studies reported “pain” as an outcome indicator and nine studies reported “anxiety” as an outcome indicator. The characteristics of the included studies are shown in Table 1.

**Table 1:** Characteristics of the studies included in the meta-analysis.

Author, year, country	Population	TBSA(%) (mean ± SD)	Age(year) (mean ± SD)	Total/M/F	Type	Procedure	Duration	Frequency	Control	Scale	Outcome
Najafi Ghezeljeh et al. (2017) Iran (36)	Adult patients	T:23.93 ± 9.547 C:23.37 ± 9.337	T:32.97 ± 8.67 C:32.23 ± 8.53	T:60/31/29 C:60/31/29	MT	No specific procedure	20min	Over 3 consecutive days	CON	VAS	①②③
Najafi Ghezeljeh et al. (2018) Iran (37)	Adult patients	T:23.93 ± 9.547 C:23.37 ± 9.337	T:32.97 ± 8.67 C:32.23 ± 8.53	T:60/31/29 C:60/31/29	MT	Before wound care	20min	Once	CON	PVoBSPAS	②
Hsu et al. (2016) China, Taiwan (35)	Adult patients	T:18.14 ± 14.32 C:17.82 ± 15.34	T:35.83 ± 13.05 C:38.41 ± 15.82	T:35/22/13 C:35/25/10	MT	Dressing change	60–90min	Once over 5 consecutive days	CON	NRS	①②
Ghezeljeh et al. (2015) Iran (34)	Adult patients	T:24.08 ± 9.68 C:23.02 ± 9.48	T:31.08 ± 8.11 C:31.18 ± 8.43	T:50/31/19 C:50/31/19	MT	No specific procedure	20min	Once over 3 consecutive days	CON	VAS	①②③④
Rohilla et al. (2018) India (38)	Adult patients & Adolescent patients	Total:27 ± 19	Total:28.8 ± 13.7	Total:10/8/2	MT	Dressing change	30 min before dressing change to 30 min after dressing change	Once a day for 2 months	CON	NRS/STAT	①②
Eid et al. (2021) Egypt (39)	Adolescent patients	Less than 20%	T:9.83 ± 1.25 C:9.71 ± 1.16	T:15/8/7 C:15/6/9	MT	Undergo rehabilitation training	15min	3 times weekly for12 successive weeks	CON	VAS	①
Yaghob M et al. (2022) Iran (40)	Older Outpatients	T:76.79 ± 8.4 C:62.45 ± 8.3	T:66.07 ± 5.24 C:67.56 ± 5.02	T:45/NA/NA C:45/NA/NA	MT	Dressing change	20 min before dressing change until the end of dressing change	Once	CON	BSPAS/VAS	①②
Kahar et al. (2010) Singapore (31)	Adult patients	T:<30% C:<20%	T:21∼60 C:21∼60	T:15/12/3 C:15/14/1	MT	Dressing change	NA	Once a day for 2 months	CON	NRS	①
Najafi Ghezeljeh et al. (2016) Iran (32)	Adult patients	T:23.64 ± 9.49 C:23.45 ± 9.52	T:30.31 ± 8.14 C:32.33 ± 8.87	T:55/36/19 C:55/3124	MT	Dressing change	20 min before dressing change until the end of dressing change	Once a day for three days	CON	VAS	①②③
Shoghi et al. (2022) Iran (33)	Child patients	Total:9∼35%	T:4.23 ± 1.30 C:4.30 ± 1.36	T:40/16/24 C:40/22/18	MT	Dressing change	20 min before dressing change until the end of dressing change	Once	CON	VAS/OBSD-R	①②
Konstantatos et al. (2009) Australia (41)	Adult patients	T:15.5 ± 14.8 C:15.1 ± 10.7	T:36.1 ± 15.7 C:41.1 ± 16.2	T:43/NA/NA C:43/NA/NA	VR	Dressing change	Before dressing change	Once	CON	VAS	①
Kipping et al. (2012)Australia (42)	Adolescent patients	T: 5.1 ± 6.3 C: 4.7 ± 4.5	T:12.6 ± 1.3 C:13.5 ± 1.8	T:20/13/7 C:21/15/6	VR	Dressing change	A few minutes before dressing change until the end of dressing change	Once	CON	VAS/FLACC	①
Hoffman et al. (2020) America (43)	Child patients	44 (16–86)	(6–17)	T:25/NA/NA C:25/NA/NA	VR	Dressing change	NA	Once	CON	VAS	①
Ail et al. (2021) Egypt (45)	Child patients	T:21.55 ± 3.29 C:20.82 ± 2.52	T:13.82 ± 1.4 C:12.55 ± 2.06	T:11/7/4 C:11/6/5	VR	Undergo rehabilitation training	Several minutes before beginning the exercises and until the end of the session	Once	CON	VAS	①
Blokzijl et al. (2023) Netherlands (46)	Adult patients & Adolescent patients	T:4.9 ± 2.8 C:5.0 ± 4.4	T:40.1 ± 21.2 C:29.2 ± 12.9	T:21/15/6 C:17/15/2	VR	Dressing change	3–6min(during in the dressing change)	Once	CON	VAT	①
Kaya et al. (2023) Turkey (47)	Child patients	T:5.88 ± 2.8 C:5.03 ± 2.7	T:8.67 ± 1.48 C:8.66 ± 1.40	T:33/15/18 C:32/14/18	VR	Dressing change	Several minutes before dressing change until the end of dressing change	Once	CON	Wong–Baker FACES Pain Rating Scale/STAI-C	①②
Joo et al. (2020) Korea (44)	Adult patients	T:27.71 ± 20.15 C:27.38 ± 20.65	T:48.07 ± 8.14 C:41.69 ± 14.05	T:28/28/0 C:29/26/3	VR	Undergo rehabilitation training	30min	Once a day for 4 weeks	CON	MHQ	①

Outcome:①=Pain ②=Anxiety ③=Relaxation ④=Basic vital signs. CON, control group with routine care; MT, music therapy; VR, virtual reality; T, experimental group; C, control group; NA, unavailable; VAS, visual analogue scale; STAI, Spielberger State Trait Anxiety Inventory; BSPAS, burn-specific pain anxiety scale; PVo-BSPAS, persian version burn-specific pain anxiety scale; NRS, numeric rating scale; FLACC, faces, legs, activity, cry and consolability(Pain scores for 3–4 year olds and non-verbalising children; FPS-R, faces pain scale-revised (pain scores for verbalising 4–8 years old), VAT, visual analogue thermometer, standard clinical assessment; STAI-C, state-trait anxiety inventory for children; MHQ, Michigan Hand Outcomes Questionnaire (MHQ); OBSD-R, observational scale of behavioral distress-revised.

### 3.4 Network meta-analysis

#### 3.4.1 Pain

**Figure 3:**
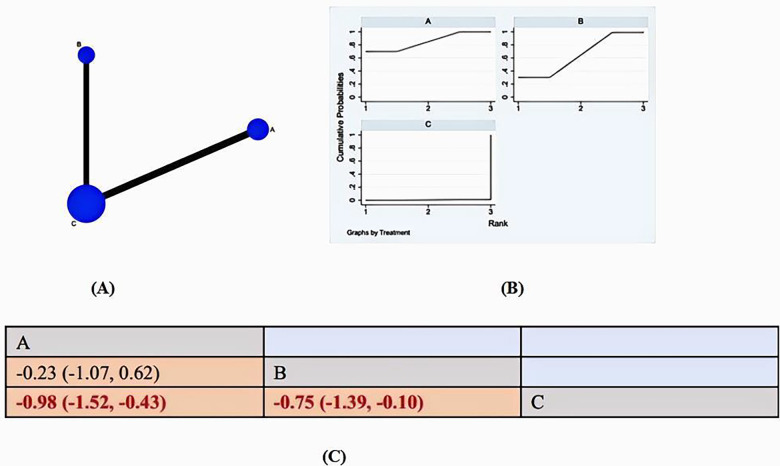
**(A)** NMA figure for PAIN. **(B)** SUCRA plot for PAIN. **(C)** Pain League chart.

The literature review included 16 studies that reported on pain. Of these, 9 studies (31–36, 38–40) used music therapy as the intervention, while 7 studies (41–47) used virtual reality. All 16 reports provided data that was suitable for statistical analysis. Therefore, 16 studies with 1009 patients and 3 pharmacological treatments are reported. Figure 3A reports the network plot for the 2 treatment classes analyzed. The main results are reported in Figure 3B and Figure 3C: SUCRA and Pain League chart.

All of the *p*-values for the indirect and direct comparisons between all of the studies were tested for consistency and inconsistency, and all of the *p*-values were greater than 0.05, indicating that the effect of consistency between the studies was acceptable. (Supplementary Table S2)

The results of the network meta-analysis showed that relative to the control groups’ routine measures, music therapy [MD = 0.98, 95% CI = (1.52, 0.43)], VR [MD = 0.75, 95% CI = (−1.39, 0.10)] were superior to the control group in reducing burn patients’ pain, the details of which are shown in Figure 3C. Compared to the control group, both music therapy and VR were found to significantly reduce pain in burn patients. The SUCA results show the following probability-based ranking for the efficacy of the two interventions in reducing burn patients’ pain: MT(85.4%)>VR(64%)>CON (0.6%). Music therapy is considered the most effective method for reducing pain in burn victims.

#### 3.4.2 Anxiety

**Figure 4:**
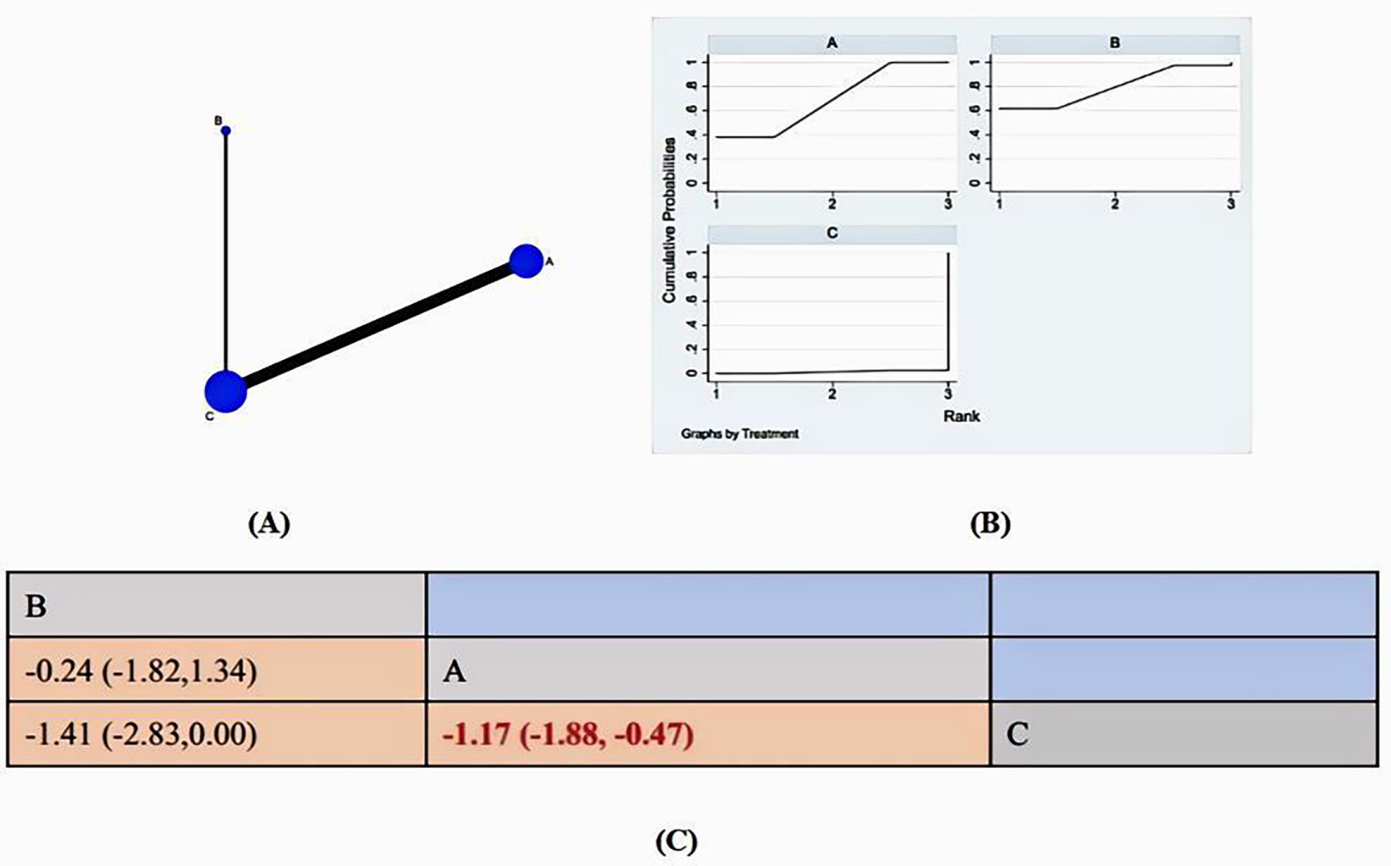
**(A)** NMA figure for anxiety. **(B)** SUCRA plot for Anxiety. **(C)** Anxiety League chart.

The literature review included 9 studies that reported on pain. Of these, 8 studies (32–38, 40) used music therapy as the intervention, while 1studies (47) used virtual reality. All 9 reports provided data that was suitable for statistical analysis. Therefore, 9 studies with 865 patients and 2 treatments are reported. Figure 4A reports the network map for the 2 treatment classes analyzed. The main results are reported in Figures 4B,C: SUCRA and Anxiety League chart.

All of the *p*-values for the indirect and direct comparisons between all of the studies were tested for consistency and inconsistency, and all of the *p*-values were greater than 0.05, indicating that the effect of consistency between the studies was acceptable (Supplementary Table S2).

The network meta-analysis results indicate that music therapy [MD = −1.17, 95% CI = (−1.18, −0.47)] was more effective in reducing pain in burn patients than conventional measures in the control group. Although VR [MD = −1.41, 95% CI = (−2.83,0.00)] was included, the confidence interval was at the zero boundary, suggesting that it may be related to the inclusion of less relevant literature, the details of which are shown in Figure 4C. Compared to the control group, both music therapy and VR were found to significantly reduce anxiety in burn patients. The SUCA results show the following probability-based ranking for the efficacy of the two interventions in reducing burn patients’ anxiety: VR(79.5%)>MT(69.2%)>CON (1.3%). VR therapy is considered the most effective method for alleviating anxiety in burn victims.

### 3.5 Publication bias test

**Figure 5:**
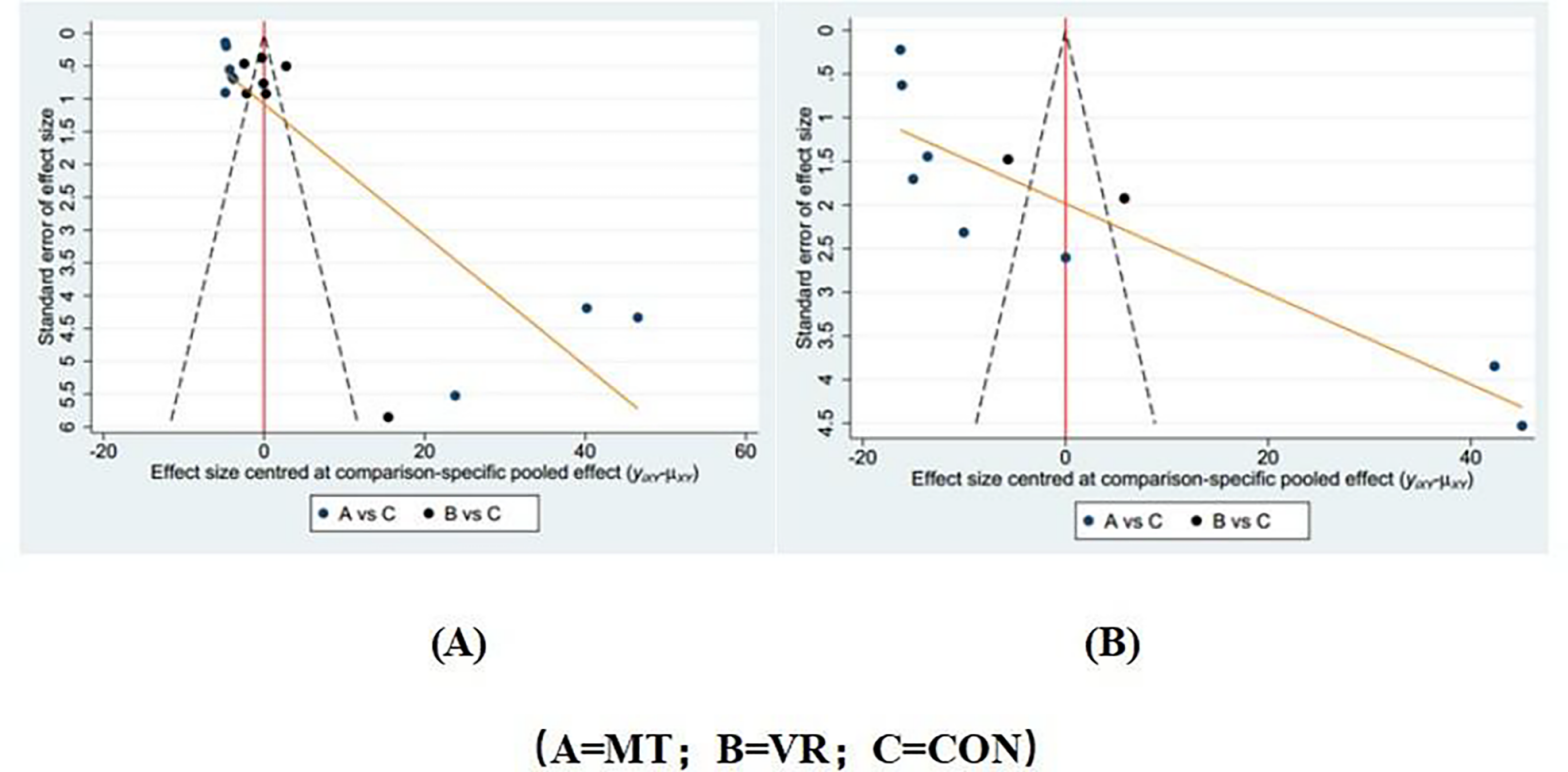
Funnel plot on publication bias. **(A)** Pain; **(B)** Anxiety.

Separate funnel plots were constructed for all outcome indicators to test for possible publication bias. Visual inspection of the funnel plots did not reveal any significant publication bias (48). Details as shown in Figure 5.

### 3.6 Other results

Analysis of other expected outcomes, such as relaxation and basic vital signs (including heart rate, respiratory rate and blood pressure) was unsuccessful due to lack of measurement or insufficient data in the manuscripts.

## 4 Discussion

### 4.1 Pain

Regarding pain reduction, our study (as shown in the SUCRA results) showed that both music therapy and VR were effective in relieving pain in burn patients, but music therapy had the advantage in significantly relieving pain, a result consistent with previous studies (34–36, 38–43, 45–47), These studies found that patients who received the intervention had significantly lower pain scores than those who did not receive such intervention. Although Konstantatos et al. 41 found that virtual therapy was an effective means of relieving patients’ pain, through our multifaceted comparison, we found that music therapy may be a more effective tool relative to virtual reality. Our study provides an evidence base for the effectiveness of non-pharmacological interventions such as VR and music therapy in managing pain.

Acute pain is a frequent challenge in healthcare, especially for burn patients, who endure significant pain with significant physical and psychological consequences. Currently, the main clinical strategy for treating this type of pain is pharmacologic analgesia (8). However, it is worth noting that long-term use of analgesics has certain side effects, and continued use will reduce the patient's tolerance to such drugs, thus reducing their efficacy. Considering the special characteristics of burn patients (who may frequently change dressings or undergo debridement surgery) (49), some methods with relatively minor side effects should be explored and found. Interventions such as music therapy and virtual reality also offer proven methods for relieving acute pain. Our study highlights the potential of these methods to provide effective analgesia while reducing the side effects and risks of medical treatments, providing patients with more treatment options and advocating for improved burn patient care. Towards more comprehensive, patient-centered pain management strategies.

### 4.2 Anxiety

According to SUCRA results, virtual reality technology is the most effective intervention for improving anxiety in burn patients. However, it is worth noting that the 95% confidence interval (CI) of the research results we obtained includes 0. The possible reason is that the sample size is small, so more subsequent RCTs are needed to strengthen the conclusions we obtained.

Our research aligns with earlier findings that show music can help patients feel less anxious and more relaxed (34–36, 38–40). While music has been looked at a lot, not many studies have explored how virtual reality can help burn patients deal with anxiety (41–43, 45–47). However, a handful of studies—including one with kids who have burn injuries—point to virtual reality being a good way to cut down on anxiety in pediatric patients (47). This backs up what we found in our research, showing that virtual reality could be a way to help ease anxiety for patients during their burn treatment.

Anxiety is often linked to the pain experienced during wound healing and after burns. Pain can also cause stress and anxiety, which in turn can worsen the pain (50). Our study contributes to the understanding of this complex relationship by showing that both music and virtual reality technology can serve as effective distractive interventions during dressing changes and treatment, thereby reducing pain and inducing relaxation (51).

### 4.3 Basic vital signs

Owing to the constraints posed by our modest sample size, we made the decision not to delve into an analysis of vital signs. Nonetheless, aligning with Najafi et al.'s research, it appears that music intervention could be more attuned to influencing respiratory rate specifically, rather than casting a wide net across all vital signs—including pulse rate, blood pressure (both systolic and diastolic), and a spectrum of other physiological metrics—reference 34. Hopefully, more data will be available in the future to support this conclusion, and perhaps these findings can serve as a basis for future discussions and research to explore the subtle effects of music on the physiological responses of burn patients.

## 5 Strengths and limitations

Our study conducted an extensive literature search and included as many relevant and eligible literature as possible. A total of 17 studies involving 1,119 patients were included, providing a relatively large sample size to reduce publication bias. The Cochrane Library Handbook was strictly followed to minimize relevant bias. In this study, we compared two common clinical non-drug treatments, music therapy and VR, to provide updated and comprehensive evidence-based recommendations.

However, based on basic research, our study has some limitations. This study only analyzed some outcome indicators, such as the length and frequency of intervention and the stage of intervention, and factors such as the patient's age, gender, and burn area were not included in the analysis. In addition, regarding the observation indicator of “anxiety”, there are relatively few literatures on virtual reality, and more RCTs are needed to support our conclusions.

Finally, readers should be cautious when interpreting our results. There are still few clinical studies that meet the inclusion and exclusion criteria, the sample size is insufficient, and the possibility of false positive rate is high. In addition, there is limited evidence for direct comparison of certain interventions. It is hoped that relevant clinical research will be expanded to improve the accuracy of the results.

## 6 Conclusions

In our study of the treatment process for burn patients, we recommend music therapy for the relief of pain and VR therapy for the relief of anxiety.

In the treatment of burn patients, it is important to consider not only the ranking results but also the absolute risk differences between interventions. Therefore, a comprehensive assessment should be conducted, taking into account the relevant conditions of the patients, in order to develop individualized treatment plans.

## Data Availability

The original contributions presented in the study are included in the article/Supplementary Material, further inquiries can be directed to the corresponding author.
