# Supplementary material for: Effects of two non-drug interventions on pain and anxiety in the nursing process of burn patients: a literature review with meta-analysis
Source: Front Rehabil Sci. 2024 Oct 29;5:1479833. doi: 10.3389/fresc.2024.1479833 (PMC11554659; doi:10.3389/fresc.2024.1479833)
Supplement: Supplementary file 1 [file Datasheet1.docx]

**Supplemental Table 1: Electronic search strategy**

**1.1 *Database:*** *PubMed <inception to Nov 24, 2023>*

| **Search** | **Query** | **Results** |
| --- | --- | --- |
| #5 | Search：**#1 AND #4** | 31 |
| #4 | Search：**(((music therapy[MeSH Major Topic]) OR (Therapy, Music[Title/Abstract])) OR (music intervention[Title/Abstract])) OR (music medicine[Title/Abstract])** | 4,131 |
| #3 | Search：**#1 AND #2** | 47 |
| #2 | Search：**((((((((((((Virtual Reality[MeSH Major Topic]) OR (Reality, Virtual[Title/Abstract])) OR (Virtual Reality, Educational[Title/Abstract])) OR (Educational Virtual Realities[Title/Abstract])) OR (Educational Virtual Reality[Title/Abstract])) OR (Reality, Educational Virtual[Title/Abstract])) OR (Virtual Realities, Educational[Title/Abstract])) OR (Virtual Reality, Instructional[Title/Abstract])) OR (Instructional Virtual Realities[Title/Abstract])) OR (Instructional Virtual Reality[Title/Abstract])) OR (Realities, Instructional Virtual[Title/Abstract])) OR (Reality, Instructional Virtual[Title/Abstract])) OR (Virtual Realities, Instructional[Title/Abstract])** | 7,622 |
| #1 | Search：**((((((((((burns[MeSH Major Topic]) OR (burn[Title/Abstract])) OR (burn complication[Title/Abstract])) OR (burn injury[Title/Abstract])) OR (burn trauma[Title/Abstract])) OR (burn wound[Title/Abstract])) OR (burning[Title/Abstract])) OR (deep burn[Title/Abstract])) OR (skin burn[Title/Abstract])) OR (thermal burn[Title/Abstract])) OR (third degree burn[Title/Abstract])** | 92,943 |

**1.2 *Database:*** *EMBASE<inception to Nov 24, 2023>*

| **History** |  | **Results** |
| --- | --- | --- |
| #11 | #3 AND #9 | 71 |
| #10 | #3 AND #6 | 253 |
| #9 | #7 OR #8 | 9,887 |
| #8 | **'therapy, music'**:ab,ti OR **'music therapy'**:ab,ti OR **'music medicine'**:ab,ti OR **'music intervention'**:ab,ti | 5,207 |
| #7 | **'music therapy'**/exp | 9,182 |
| #6 | #4 OR #5 | 32,644 |
| #5 | **'virtual reality'**:ab,ti OR **'reality, virtual'**:ab,ti OR **'virtual reality, educational'**:ab,ti OR **'educational virtual realities'**:ab,ti OR **'educational virtual reality'**:ab,ti OR **'reality, educational virtual'**:ab,ti OR **'virtual realities, educational'**:ab,ti OR **'virtual reality, instructional'**:ab,ti OR **'instructional virtual realities'**:ab,ti OR **'instructional virtual reality'**:ab,ti OR **'realities,** instructional **virtual'**:ab,ti OR **'reality, instructional virtual'**:ab,ti | 20,686 |
| #4 | 'virtual reality'/exp | 27,282 |
| #3 | #1 OR #2 | 151,111 |
| #2 | **'burn complication'**:ab,ti OR **'burn injury'**:ab,ti OR **'burn trauma'**:ab,ti OR **'burn wound'**:ab,ti OR **'burning'**:ab,ti OR **'burns'**:ab,ti OR **'deep burn'**:ab,ti OR **'skin burn'**:ab,ti OR **'thermal burn'**:ab,ti OR **'third degree burn'**:ab,ti OR **'burn'**:ab,ti | 121,870 |
| #1 | 'burn'/exp | 91,914 |

**1.3 *Database:*** *Web of Science<inception to Nov 24, 2023>*

|  | **Export** | **Results** |
| --- | --- | --- |
| #5 | #1 AND #4 | 121 |
| #4 | **(((ALL=(music therapy)) OR ALL=( Therapy, Music)) OR ALL=( music intervention)) OR ALL=( music medicine)** | 14,997 |
| #3 | #1 AND #2 | 296 |
| #2 | **((((((((((((ALL=( Virtual Reality)) OR ALL=( Reality, Virtual)) OR ALL=( Virtual Reality, Educational)) OR ALL=( Educational Virtual Realities)) OR ALL=( Educational Virtual Reality)) OR ALL=( Reality, Educational Virtual)) OR ALL=( Virtual Realities, Educational)) OR ALL=( Virtual Reality, Instructional)) OR ALL=( Instructional Virtual Realities)) OR ALL=( Instructional Virtual Reality)) OR ALL=( Realities, Instructional Virtual)) OR ALL=( Reality, Instructional Virtual)) OR ALL=( Virtual Realities, Instructional)** | 40154 |
| #1 | **(((((((((((ALL=(burns)) OR ALL=(Burn)) OR ALL=(burn complication)) OR ALL=( burn injury)) OR ALL=(burn trauma)) OR ALL=( burn wound)) OR ALL=(burning)) OR ALL=(burns)) OR ALL=(deep burn)) OR ALL=(skin burn)) OR ALL=(****thermal burn)) OR ALL=(third degree burn)** | 208,976 |

**1.4 *Database:*** *Cochrane Library<inception to Nov 24, 2023>*

|  |  | **Results** |
| --- | --- | --- |
| #1 | MeSH descriptor: [Burns] explode all trees | 2141 |
| #2 | burn OR burn complication OR burn injury OR burn trauma OR burn wound OR burning OR deep burn OR skin burn OR thermal burn OR third degree burn):ti,ab,kw | 11443 |
| #3 | #1 AND #2 | 11692 |
| #4 | MeSH descriptor: [Virtual Reality] explode all trees | 1022 |
| #5 | (Reality, Virtual OR Virtual Reality, Educational OR Educational Virtual Realities OR Educational Virtual Reality OR Reality, Educational Virtual OR Virtual Realities, Educational OR Virtual Reality, Instructional OR Instructional Virtual Realities OR Instructional Virtual Reality OR Realities, Instructional Virtual OR Reality, Instructional Virtual OR Virtual Realities, Instructional):ti,ab,kw | 6283 |
| #6 | #4 AND #5 | 6286 |
| #7 | MeSH descriptor: [Music Therapy] explode all trees | 1245 |
| #8 | (Therapy, Music OR music intervention OR music medicine):ti,ab,kw | 5501 |
| #9 | #7 OR #8 | 5501 |
| #10 | #3 AND #6 | 163 |
| #11 | #3 AND #9 | 77 |

**Supplemental Table 2: Consistency test**

**2.1** Consistency test for Pain.

|  | Direct |  | Indirect |  | Difference |  |  |  |
| --- | --- | --- | --- | --- | --- | --- | --- | --- |
|  | Coef. | Std. Err. | Coef. | Std. Err. | Coef. | Std. Err. | P>\|z\| | tau |
| A vs C | 0.9775141 | 0.2705958 | 0.4677443 | 129.0622 | 0.5097698 | 129.0625 | 0.997 | 0.756541 |
| B vs C | 0.9372828 | 0.3487294 | 1.957667 | 257.9311 | -1.020384 | 257.9313 | 0.997 | 0.7565409 |

**2.2** Consistency test for Pain.

|  | Direct |  | Indirect |  | Difference |  |  |  |
| --- | --- | --- | --- | --- | --- | --- | --- | --- |
|  | Coef. | Std. Err. | Coef. | Std. Err. | Coef. | Std. Err. | P>\|z\| | tau |
| A vs C | 1.173426 | 0.360613 | 0.7151493 | 223.7896 | 0.4582768 | 223.7896 | 0.998 | 0.9843113 |
| B vs C | 1.41446 | 0.7217042 | 2.331051 | 446.7474 | -0.9165907 | 446.7474 | 0.998 | 0.9843112 |
